# Exogenous Selenoprotein V Induces Apoptosis in Murine Testicular Teratoma Cells via Mitochondrial Dysfunction and ROS Overproduction

**DOI:** 10.3390/biom15121733

**Published:** 2025-12-12

**Authors:** Egor A. Turovsky, Elena G. Varlamova

**Affiliations:** Institute of Cell Biophysics of the Russian Academy of Sciences, Federal Research Center “Pushchino Scientific Center for Biological Research of the Russian Academy of Sciences”, 142290 Pushchino, Russia

**Keywords:** SELENOV, apoptosis, mitochondrial depolarization, ROS, calcium signaling, cell migration, cancer therapy

## Abstract

This study explores the effects of exogenous SELENOV on cellular migration, viability, mitochondrial function, ROS production, and Ca^2+^ signaling in mouse fibroblast L-929 and testicular teratoma F-9 cells. In scratch assays, 50–100 µg/mL SELENOV significantly inhibited F-9 cell migration after 48 h, while in L-929 fibroblasts, only 100 µg/mL had a suppressive effect. Viability assays revealed strong cytotoxicity in F-9 cells. Critically, at a dose of 50 µg/mL (where the corresponding volume of solvent buffer alone was non-toxic), SELENOV reduced survival to 19%, triggering late apoptosis in 76% of cells, whereas in L-929 cells, comparable effects required 100 µg/mL. Mitochondrial depolarization (JC-1/Rhodamine-123 assays) was pronounced in F-9 cells even at 50 µg/mL, while L-929 cells responded only to 100 µg/mL. Similarly, 50 µg/mL SELENOV induced significant ROS overproduction in F-9 but not in L-929 cells, correlating with upregulated NOX1, NOX4, GPX3, and GPX4 expression. Ca^2+^ imaging showed dose-dependent [Ca^2+^]ᵢ elevation, with 50 µg/mL SELENOV inducing a sustained rise in F-9 cells, whereas L-929 cells required higher doses. Strikingly, 50 µg/mL SELENOV in F-9 cells downregulated BCL-2 and BCL-xL while upregulating pro-apoptotic BAX and PUMA, suggesting selective activation of intrinsic apoptosis. These results demonstrate that F-9 cancer cells are significantly more sensitive to SELENOV than normal fibroblasts, with 50 µg/mL sufficient to trigger mitochondrial dysfunction, oxidative stress, and apoptosis. The findings highlight SELENOV’s potential as a targeted anticancer agent, particularly for germ cell tumors.

## 1. Introduction

Selenoprotein V (SELENOV) represents one of the least characterized members of the mammalian selenoprotein family. Unlike most selenoproteins, SELENOV demonstrates restricted phylogenetic distribution and is primarily expressed in testes, suggesting a specialized role in reproductive tissues [1,2]. While global Selenov knockout studies have implicated this protein in energy metabolism and selenium homeostasis [3,4], its specific cellular functions remain largely unexplored. Altered expression of various selenoproteins, including SELENOV, has been documented in a range of tumor cell lines [5], suggesting their potential involvement in oncogenesis.

The well-established involvement of selenoproteins in regulating redox balance and apoptosis [6,7], combined with SELENOV’s testis-predominant expression pattern, led us to hypothesize that it might exhibit selective bioactivity against germ cell-derived malignancies. Testicular teratomas represent an important model for investigating novel therapeutic approaches, as current treatments, while often effective, can lead to significant long-term side effects [8].

Previous studies have demonstrated that certain selenoproteins and selenium-containing compounds can induce selective cytotoxicity in cancer cells through mechanisms involving mitochondrial dysfunction, reactive oxygen species (ROS) overproduction, and calcium signaling disruption [9,10]. However, whether SELENOV possesses similar properties remains completely unknown.

Based on these considerations, we specifically investigated (1) whether recombinant SELENOV exhibits selective toxicity against mouse testicular teratoma (F-9) cells compared to normal fibroblasts (L-929); (2) whether this selectivity involves differential effects on mitochondrial function and ROS production; and (3) whether SELENOV triggers intrinsic apoptosis in a cell-type-specific manner. The cell models for this study were selected to specifically test the hypothesis of tissue-selective and transformation-dependent cytotoxicity. The F-9 murine testicular teratoma cell line was chosen as it represents a malignancy derived from the tissue with the highest endogenous expression of SELENOV—the testes. This makes it a highly relevant model for investigating whether the protein exhibits preferential activity against cancers originating from its native expression site. In contrast, L-929 murine fibroblasts served as a normal, non-testicular control cell type. Fibroblasts are a ubiquitous stromal component with fundamentally different physiology and are not a primary site of SELENOV expression. This experimental design allows for a direct assessment of whether SELENOV’s effects are specific to a cancer type from its tissue of origin versus a normal cell type from a different tissue context. Our study provides the first functional characterization of SELENOV’s potential anticancer properties, focusing specifically on its effects on cell viability, migration, mitochondrial membrane potential, ROS generation, and calcium homeostasis.

## 2. Materials and Methods

### 2.1. Cloning and Expression of Recombinant mSELENOV (Sec → Cys)

To obtain recombinant mSELENOV (Sec → Cys), total RNA was first isolated from mouse testes and reverse transcription was performed using the methods described above. For cloning the mSELENOV open reading frame (ORF) into the pET23b+ vector (Novagene, Sacramento, CA, USA), the following primers were used: 5′-CTACGAATTCATGAATAACAAGGCGCGGGTC-3′ and 5′-ATCACTCGAGCCTCTTCTTGATCTCTTCGTC-3′.

The primers were designed to incorporate a 6xHis-tag at the C-terminus of recombinant mSELENOV. To generate recombinant mSELENOV (Sec → Cys), the TGA codon was replaced with TGT using a pair of overlapping primers: 5′-ATGTACTGTGGCCTCTGTAGCTATGGCCTTCGGTAC-3′ and 5′-ACCGAAGGCCATAGCTACAGAGGCCACAGTAC-3′.

This substitution of selenocysteine (Sec)-encoding TGA with cysteine (Cys)-encoding TGT was necessitated by the peculiarities of mammalian selenoprotein biosynthesis, as previously described in detail [11,12,13,14,15].

Expression of the resulting genetic construct was performed in the bacterial system *E. coli* strain Rosetta, with induction of the lac operon using 0.5 mM IPTG at 37 °C for 3 h.

### 2.2. Isolation and Purification of mSELENOV (Sec → Cys)

Recombinant protein was isolated from bacterial cells (*E. coli*) under native conditions. Cells were resuspended in lysis buffer (50 mM NaH_2_PO_4_, 300 mM NaCl, 10 mM imidazole, supplemented with 1 mg/mL lysozyme and 1 mM PMSF, pH 8.0) and incubated for 30 min at 4 °C. Cell lysis was completed by ultrasonic disruption at 22 kHz for 15 min at 4 °C. The lysate was centrifuged at 16,880× *g* for 20 min and filtered through 0.2 μm sterile filters (GVS, Sanford, USA).

Protein purification was performed using nickel-agarose affinity chromatography. Lysates were bound to nickel-agarose for 2 h at 4 °C. Non-specifically bound proteins were removed by extensive washing with buffer (50 mM NaH_2_PO_4_, 300 mM NaCl, 20 mM imidazole, pH 8.0). Purified mSELENOV (Sec → Cys) was eluted with elution buffer (50 mM NaH_2_PO_4_, 300 mM NaCl, 250 mM imidazole, pH 8.0). The protein solution was dialyzed with stepwise imidazole reduction. The final purified mSELENOV (Sec → Cys) in storage buffer (50 mM NaH_2_PO_4_, 300 mM NaCl, pH 8.0) was added to cell monolayers at 50 μg/mL concentration. Control samples received equivalent volumes of buffer (50 mM NaH_2_PO_4_, 300 mM NaCl, pH 8.0). Our cloning procedure, expression optimization, and recombinant mSELENOV (Sec → Cys) purification have been previously demonstrated in our earlier works [2,16,17]. The purity and identity of the recombinant mSELENOV (Sec → Cys) were verified by SDS-PAGE (12.5% polyacrylamide gel) under reducing conditions and Coomassie Brilliant Blue R-250 staining. A single dominant band corresponding to the expected molecular weight of the His-tagged protein was observed in the purified fraction. Densitometric analysis of the gel confirmed a purity of >90%. The presence of the protein in both soluble and insoluble fractions after cell lysis was consistent with efficient expression without significant inclusion body formation, as confirmed by gel analysis (Supplementary Figure S1 for representative gel image). The concentration of recombinant SELENOV is reported in µg/mL throughout this study. For informational purposes, based on the calculated molecular weight of the monomeric protein (Da), the doses used (10, 50, and 100 µg/mL) correspond approximately to 0.4, 2, and 4 µM, respectively. It should be noted that the actual molar concentration of biologically active units may differ depending on the protein’s oligomeric state and functional homogeneity in solution.

### 2.3. Cell Culture

The study utilized several established cell lines: F9 cells (a murine embryonal carcinoma model derived from testicular teratoma of C57BL/6 mice) and mouse fibroblasts (L-929), all obtained from the American Type Culture Collection (ATCC, Manassas, VA, USA). Cells were maintained in DMEM supplemented with 10% fetal bovine serum (FBS) and gentamicin as an antimicrobial agent. Cells used in all experiments were within passage numbers 4 to 12. Prior to experiments, cells were seeded onto round coverslips and cultured for 48 h until reaching 80–95% confluency. Cells were tested for mycoplasma contamination using the MycoAlert™ Mycoplasma Detection Kit (Cat# LT07-318, Lonza, Basel, Switzerland) according to the manufacturer’s protocol. All experiments were performed with mycoplasma-free cells. Volume-matched buffer controls were included for SELENOV doses of 50 μg/mL and 100 μg/mL. The 10 μg/mL SELENOV dose was compared to the untreated control, as the delivered buffer volume (20 μL/mL) was considered negligible. The protein solvent buffer (50 mM NaH_2_PO_4_, 300 mM NaCl, pH 8.0) was used to deliver SELENOV at high concentrations. Our initial experiments revealed that the buffer volumes required for the 50 µg/mL (100 µL/mL) and 100 µg/mL (200 µL/mL) SELENOV treatments induced significant cellular stress on their own (see Results, Section 3.1 and Section 3.2). Therefore, for all experiments involving these doses, the equivalent volume of solvent buffer was used as the primary control group for statistical comparison to isolate the effects attributable specifically to the SELENOV protein from those caused by the vehicle.

### 2.4. Wound Healing Assay

Cell migration was assessed using a standardized wound healing assay. Cells were seeded in 24-well plates (at a density of 1.0 × 10^5^ cells per well) and cultured until reaching 90% confluence under normal growth conditions. A uniform linear scratch was created in the cell monolayer using a sterile 200 μL pipette tip. After wounding, wells were gently washed with phosphate-buffered saline (PBS) to remove detached cells, followed by replacement with low-serum DMEM (2% FBS) to minimize proliferation effects. Experimental treatments consisting of various SELENOV concentrations or equivalent volumes of protein solvent buffer were then added to the cultures.

Initial wound images (0 h baseline) were acquired using an Axiovert 200 M inverted microscope (Carl Zeiss) equipped with a 10× objective and AxioCam HSm digital camera. Following treatment, plates were returned to the incubator for continued culture under standard conditions. Cell migration was documented by capturing images at 24 h and 48 h post-treatment. Quantitative analysis of wound closure was performed using the Wound Healing Size Tool plugin in ImageJ software (available at: https://github.com/AlejandraArnedo/Wound-healing-size-tool, accessed on 9 November 2025), which provided precise measurements of remaining wound area at each time point. The threshold for image binarization and wound region extraction was set automatically using the built-in Otsu’s algorithm (Otsu’s method), eliminating subjective manual selection. This threshold was applied consistently and uniformly to all images within a single experiment. Data are from three independent biological replicates (*n* = 3), each performed in duplicate technical replicates.

### 2.5. Assessment of Cell Death via Fluorescent Staining

Cells were plated on 25 mm round coverslips at a density of 5.0 × 10^4^ cells per coverslip. Cell death was assessed using dual fluorescent staining with Hoechst 33342 (2 μM) and propidium iodide (1 μM). Following a 15-min incubation in HBSS at 37 °C, cells were washed to remove excess dye. Hoechst 33342 uniformly stains all nuclei but shows 3–4-fold higher fluorescence intensity in apoptotic cells due to chromatin condensation, while propidium iodide specifically labels nuclei of necrotic cells with compromised membranes.

Imaging was performed using a Zeiss Axio Observer Z1 inverted microscope configured with: Filter Set 01 (Ex365/12 nm, Em LP397 nm) for Hoechst 33342 and Filter Set 20 (Ex546/12 nm, Em575–640 nm) for propidium iodide detection. A 20× immersion objective (NA 0.70) was used for all acquisitions. Cell populations were categorized as: viable (normal Hoechst/PI-negative), early apoptotic (enhanced Hoechst/PI-negative), late apoptotic (intense Hoechst with partial PI uptake), or necrotic (strong PI staining). Five random fields per sample were analyzed across three biological replicates (distinct passages) with three technical replicates each (*n* = 9 total per condition).

For simultaneous apoptotic/viable cell monitoring post-SELENOV treatment, we used an Annexin V-AF 488 Apoptosis Detection Kit (Lumiprobe) with propidium iodide and CytoCalcein 450. Cells were cultured in 35 mm Petri dishes to confluence before treatment with SELENOV concentrations or vehicle control. After 24-h exposure, cells were trypsinized and processed according to the manufacturer’s protocol: washed with chilled PBS (pH 7.4) and 1× binding buffer, then resuspended in cold 1× binding buffer. Aliquots (100 μL of 1 × 10^5^–1 × 10^6^ cells/mL) were incubated with 5–10 μL Annexin V-AF 488 for 10–15 min (room temperature, dark). Without washing, 400 μL 1× binding buffer was added followed by 1 μL each of propidium iodide and CytoCalcein 450 (5-min incubation, dark). Samples were maintained at 2–8 °C in darkness and analyzed within 4 h using fluorescence microscopy: apoptotic cells (FITC channel, Ex490/Em525 nm), necrotic cells (PI, Ex550/Em650 nm), and viable cells (violet channel, Ex405/Em450 nm). For analysis of images obtained with the Annexin V/PI and Hoechst 33342 kits, positive fluorescence thresholds were established based on the signal of negative controls (unstained cells) and remained constant for all samples within a single analysis run. Cells were considered Annexin V-positive or PI-positive if the fluorescence intensity exceeded the level established for the unstained control, according to the kit manufacturer’s standard protocol.

### 2.6. Measurement of ROS Production

Measurement of intracellular reactive oxygen species (ROS) production was performed using the fluorescent probe 2′,7′-dichlorodihydrofluorescein diacetate (H_2_DCF-DA). Cell lines were seeded in 96-well plates (at a density of 5.0 × 10^3^ cells per well) and cultured for 48 h to reach appropriate confluence. Following the culture period, cells were treated with various concentrations of SELENOV or equivalent volumes of protein solvent buffer for 24 h. After treatment, cells were washed with phosphate-buffered saline (PBS) and incubated with 20 μM H_2_DCF-DA in serum-free medium for 30 min at 37 °C in a 5% CO_2_ atmosphere. Fluorescence intensity was measured using a Spark 10 M multimode microplate reader (Tecan) with excitation/emission filters set at 485/535 nm. It is important to note that the H_2_DCF-DA fluorescent probe detects total intracellular ROS levels without distinguishing between mitochondrial and extramitochondrial sources. To minimize photobleaching artifacts and probe-derived ROS generation, fluorescence readings were acquired at 5-min intervals. In acute oxidative stress experiments, baseline fluorescence was established prior to administration of 10 μM antimycin A as a ROS-inducing agent. Fluorescence data were analyzed using ImageJ software (NIH) for initial processing, followed by statistical analysis and graphical representation using Origin 8.5 (OriginLab) and GraphPad Prism 8 (GraphPad Software, San Diego, CA, USA). H_2_DCF-DA fluorescence data were processed after background subtraction. The threshold for considering a positive ROS signal was set based on the average fluorescence value in control (untreated) wells and remained constant for all samples within a single experiment. All experimental results are expressed as mean ± standard error of the mean (SEM) from at least three independent biological replicates, with each replicate containing triplicate technical measurements. This method provides measurement of total intracellular ROS without discrimination between different cellular sources.

### 2.7. Total RNA Isolation and Reverse Transcription

Total RNA isolation from cell lines and testicular tissue was performed using a monophasic phenol-guanidine isothiocyanate solution ExtractRNA (#BC032, Evrogen, Russia). Total RNA was isolated from the testes of mice obtained from the tissue cryobank of our institute. The original tissues were procured in accordance with a protocol approved by the Bioethics Committee of the Institute of Cell Biophysics (Approval ID: 1/092022, date: 8 September 2022): euthanasia by cervical dislocation under isoflurane anesthesia, followed by immediate freezing of the extracted tissues in liquid nitrogen to preserve RNA integrity. Petri dishes with cell monolayers or 100 mg of testicular tissue were taken and homogenized in 1 mL of ExtractRNA, after which 200 μL of chloroform was added and centrifuged at 16,250× *g* for 30 min at room temperature. The aqueous phase was collected, 500 μL of 100% isopropanol was added, and the mixture was centrifuged at 16,250× *g* for 10 min at room temperature. To the pellet, 1 mL of 75% ethanol was added and centrifuged at 16,250× *g* for 10 min at room temperature. This procedure was repeated twice, after which the supernatant was removed, the pellet was air-dried and dissolved in nuclease-free water.

For first-strand complementary DNA synthesis from single-stranded RNA templates, the MMLV RT kit (#SK021, Evrogen, Russia) was used. The reaction was performed using oligo(dT)15 primers and MMLV reverse transcriptase. The amount of total RNA in each reverse transcription reaction was 0.5–2 μg. The resulting cDNA was used as a template for real-time PCR.

### 2.8. Design of Oligonucleotides and Real-Time PCR

Primer design used in this work was performed using Gene Runner software, Version 6.5.52 Beta (https://www.generunner.net, accessed on 9 November 2025) (Table 1). The annealing temperature for all primer pairs used in real-time PCR was 60 °C. An important condition was that forward and reverse primers were separated by one or more introns to avoid obtaining false positive results. For real-time PCR with SYBR Green I intercalating dye, we used 5xqPCRmix-HS SYBR reaction mix (#PK147, Evrogen, Russia). The reaction was performed in the following amplification mode: initial denaturation (1 cycle) 95 °C 1 min, denaturation (40 cycles) 95 °C 30 s, annealing (40 cycles) 60 °C, 30 s, elongation (40 cycles) 72 °C, 30 s. Following amplification, melt curve analysis was performed to verify the specificity of each PCR product. All reactions produced single sharp peaks, confirming the absence of primer-dimers and non-specific amplification. The change in the level of mRNA expression of the studied proteins before and after treatment was determined by the formula TUE = 2 − ΔΔCt, where ΔΔCt is the difference in ΔCt values for each gene before and after cell treatment. When performing RT-PCR, reference gene-encoding glyceraldehyde-3-phosphate dehydrogenase was used. Gene expression analysis was performed on cDNA derived from three independent biological replicates per condition.

### 2.9. Fluorescent Ca^2+^ Imaging

The intracellular calcium concentration ([Ca^2+^]_i_) was measured using Fura-2/AM fluorescence microscopy. Cells were cultured on round coverslips (25 mm round coverslips at a density of 5.0 × 10^4^ cells per coverslip) in DMEM supplemented with 10% fetal bovine serum for 48 h until reaching 80–90% confluency. For Fura-2/AM loading, cells were incubated in Hank’s Balanced Salt Solution (HBSS) containing (in mM): 156 NaCl, 3 KCl, 2 MgSO_4_, 1.25 KH_2_PO_4_, 2 CaCl_2_, 10 glucose, and 10 HEPES (pH 7.4) at 37 °C for 40 min, followed by a 15-min washout period. The final concentration of Fura-2/AM was 4–5 μM.

Measurements were performed using an inverted motorized fluorescence microscope Axiovert 200 M (Carl Zeiss Microscopy GmbH, Jena, Germany) equipped with a high-speed monochrome CCD camera AxioCam HSm (Carl Zeiss Microscopy GmbH, Germany) and a high-speed filter changer Ludl MAC 5000 (Ludl Electronic Products, Hawthorne, NY, USA). Fura-2 fluorescence was excited at 340 and 380 (387) nm, and emission was recorded at 3-s intervals. Background fluorescence was subtracted using ImageJ software (RRID: SCR_003070) with the Math Subtract plugin. The F340/F380 ratio was calculated after subtracting background fluorescence. Thresholds for determining basal calcium levels and response amplitude were established based on recordings from control cells (treated with buffer only) and were applied uniformly to all experimental groups. Data were expressed as the ratio of fluorescence intensities at 340 and 380 nm excitation wavelengths. All experiments were conducted in HBSS supplemented with 10 mM HEPES (pH 7.4) at 28 °C. Reagents were applied and washed out using a perfusion system with a flow rate of 15 mL/min. Imaging was performed using an HCX PL APO 20.0 × 0.70 IMM UV objective (refractive index 1.52). Camera settings were as follows: resolution 500 × 500 pixels (voxel size 0.724 × 0.723 μm), binning 2 × 2, and 14-bit depth. Detailed methodology can be found in references [18,19]. Experiments were repeated using cells from three independent biological replicates (different passages), with multiple cells/coverslips analyzed per replicate.

### 2.10. Assessment of Mitochondrial Membrane Potential

Measurement of ΔΨm in acute experiments was performed using the fluorescent probe Rhodamine 123. The excitation wavelength of the probe fluorescence was 488 nm, and the emission wavelength was 525 nm. The probe concentration was 1 μM. At a ratio of 1 μM probe/1 mg mitochondrial protein/1 mL, a linear dependence of changes in the probe fluorescence intensity on ΔΨm changes was observed in the range from 200 to 50 mV. At these concentrations, Rhodamine 123 did not affect oxidative phosphorylation parameters.

In the chronic experiments mitochondrial membrane potential was assessed using the JC-1 fluorescent probe, which exhibits potential-dependent accumulation in mitochondria, shifting from red fluorescence in polarized mitochondria to green fluorescence upon depolarization. Cells were treated for 24 h with either 1 μM FCCP (as a positive control) or different concentrations of SELENOV or equivalent volume of protein solvent followed by washing with HBSS and incubation with 10 μg/mL JC-1 for 30 min at 37 °C in a 5% CO_2_ atmosphere. Fluorescence imaging was performed using a Leica DMI6000B inverted microscope equipped with a HAMAMATSU C9100-13 CCD camera and Leica Ultra-Fast Filter system, maintaining constant 37 °C temperature during acquisition. Image analysis was conducted in ImageJ (version 1.52a) using background subtraction and particle analysis to determine the red-to-green fluorescence ratio as a quantitative measure of mitochondrial membrane potential. For each experimental condition, a minimum of 100 cells from five random fields were analyzed to ensure statistical significance, with FCCP serving as a positive control for mitochondrial depolarization in all experiments. The specificity of JC-1 for mitochondrial membrane potential assessment has been previously validated in multiple studies. JC-1 image analysis was performed in ImageJ after background subtraction. A semiautomated mode was used to isolate mitochondrial signals and calculate the red/green fluorescence ratio. The threshold for binarization in the green channel (the monomeric form of JC-1) was visually set based on control samples (untreated cells and cells treated with 1 μM FCCP) and then fixed for all subsequent images in the experiment. A similar procedure was used for the red channel (the aggregated form of JC-1). This ensured consistency of analysis across different experimental groups. Experiments were repeated using cells from three independent biological replicates (different passages), with multiple cells/coverslips analyzed per replicate.

### 2.11. Statistical Analysis

All data are presented as the mean ± standard error of the mean (SEM) from at least three independent biological replicates. Statistical analysis was performed using GraphPad Prism software (GraphPad Software, San Diego, CA, USA). The normality of data distribution was assessed using the Shapiro–Wilk test. For comparisons between two groups, a two-tailed unpaired or paired Student’s *t*-test was used, as appropriate. For comparisons across three or more groups, one-way analysis of variance (ANOVA) was applied, followed by Tukey’s post hoc test for multiple comparisons. Differences were considered statistically significant at *p* < 0.05.

## 3. Results

### 3.1. Effects of Exogenous SELENOV on Cell Migration

Changes in cell migration serve as a key indicator of the antitumor potential of biologically active compounds. In two-dimensional cultures of normal cells, established intercellular contacts suppress further proliferation, leading to the formation of a confluent monolayer. Notably, this contact inhibition is lost in many cancers cell cultures. This phenomenon is considered an in vitro analogue of the in vivo mechanism that maintains tissue homeostasis, which becomes disrupted during tumorigenesis.

To assess migratory capacity under controlled conditions, monolayer integrity was mechanically disrupted by creating a scratch wound using a pipette tip. Cells were treated with various concentrations of SELENOV or equivalent volumes of protein solvent buffer. Wound closure was monitored using light microscopy immediately after scratching and at 24-h intervals.

**Figure 1:**
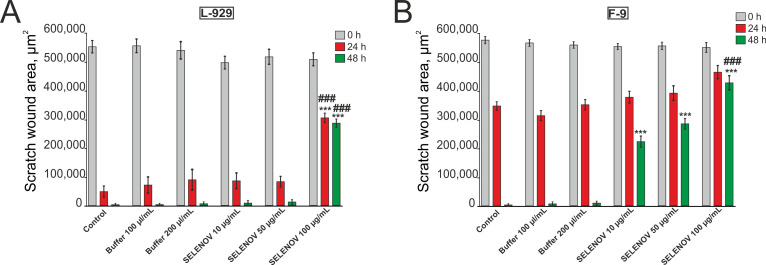
Effects of different SELENOV concentrations on the migration of mouse fibroblast L-929 cells (**A**) and F-9 testicular teratoma cells (**B**). Buffer 100 μL/mL and 200 μL/mL represent protein solvent buffer volumes corresponding to SELENOV applications at concentrations of 50 μg/mL and 100 μg/mL, respectively. The 10 μg/mL SELENOV group was compared to the untreated control, as the corresponding buffer volume was negligible. Quantification of scratch wound area (μm^2^) over time in L-929 (**A**) and F-9 (**B**) cell lines at 0, 24, and 48 h. Data represent mean ± SE, *n* = 3. Statistical significance was determined by one-way ANOVA followed by Tukey’s post hoc test. *** *p* < 0.001 versus the Control group; ### *p* < 0.001 versus the corresponding Buffer group. Representative images of cell cultures are shown in Supplementary Figures S2 and S3.

In mouse fibroblast L-929 cells, application of either solvent buffer or SELENOV at concentrations of 10 μg/mL and 50 μg/mL did not affect cell migration, with wounds completely closing within 48 h. However, treatment with 100 μg/mL SELENOV significantly inhibited cell migration after both 24-h and 48-h incubations (Figure 1A).

In F-9 testicular teratoma cells, solvent buffer application did not inhibit cell migration, and wounds healed completely after 48 h of culture. Treatment with SELENOV at concentrations of 10 μg/mL, 50 μg/mL, or 100 μg/mL showed no significant effect on cell migration after 24 h of incubation. However, after 48 h of incubation, significant suppression of migration was observed, preventing complete wound closure in the culture (Figure 1B). Notably, this suppression at both 50 µg/mL and 100 µg/mL SELENOV was evident compared to their respective buffer controls, which showed complete wound closure, indicating that the anti-migratory effect is a property of the protein itself.

### 3.2. Effects of Exogenous SELENOV on Cell Viability

**Figure 2:**
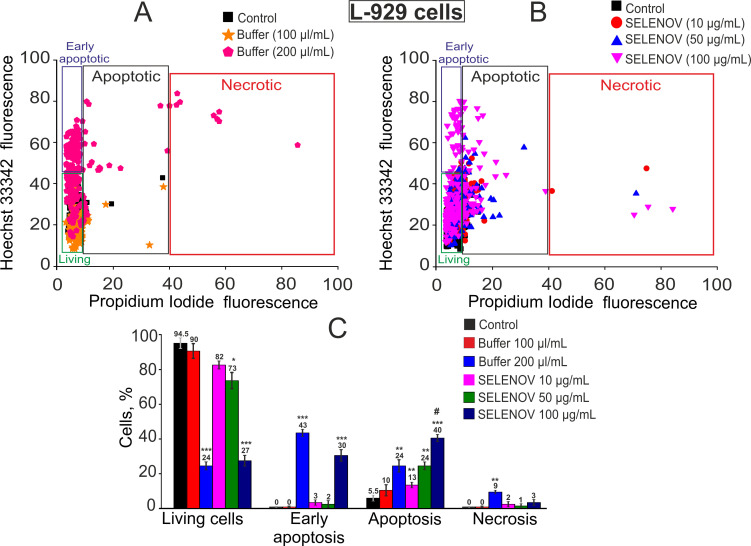
Effects of 24-h pre-incubation of L-929 cells with protein solvent buffer or various concentrations of recombinant SELENOV on cell viability. (**A**) Viability cytogram of L-929 cells following treatment with 100 μL/mL (equivalent to 50 μg/mL SELENOV application) or 200 μL/mL (equivalent to 100 μg/mL SELENOV application) of solvent buffer. The 10 μg/mL SELENOV group was compared to the untreated control, as the corresponding buffer volume was negligible. (**B**) Viability cytogram of L-929 cells after treatment with different concentrations of recombinant SELENOV. (**C**) Effects of solvent buffer or recombinant SELENOV on cell viability and induction of cell death pathways. Data are presented as mean ± SEM. Statistical significance was determined by one-way ANOVA followed by Tukey’s post hoc test. * *p* < 0.05, ** *p* < 0.01, *** *p* < 0.001 versus the Control group; # *p* < 0.05 versus the corresponding Buffer group.

In control conditions (no treatment), 94.5% of L-929 cells remained viable, with only 5.5% showing late-stage apoptosis (Figure 2A,C). Addition of 100 μL protein solvent buffer to 900 μL culture medium following 24-h pre-incubation induced apoptosis in 10% of cells, while the remainder-maintained viability. Increasing the solvent buffer volume to 200 μL (added to 800 μL culture medium) resulted in a significant reduction in viable cells to 24%, with early-stage apoptosis observed in 43% of cells, late-stage apoptosis in 24%, and necrosis in 9% of cells (Figure 2A,C).

Pre-incubation with 10 μg/mL SELENOV did not significantly reduce cell viability, with late-stage apoptosis detected in 13% of cells. Treatment with 50 μg/mL SELENOV decreased L-929 cell viability to 73%, inducing late-stage apoptosis in 24% of cells. Exposure to 100 μg/mL SELENOV for 24 h reduced cell viability to 27%, triggering early-stage apoptosis in 30% of cells and late-stage apoptosis in 40% of cells (Figure 2B,C). It is important to note that this represents an additive effect on top of the substantial baseline toxicity already established by the 200 µL/mL solvent buffer control (which reduced viability to 24%).

**Figure 3:**
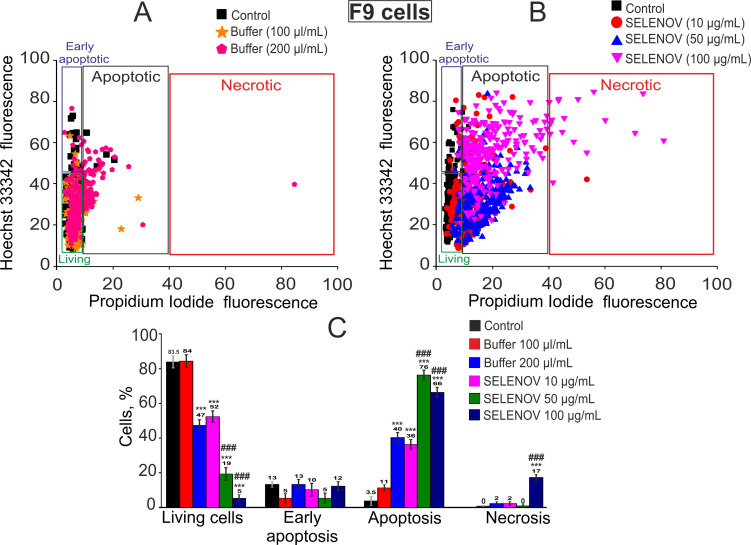
Effects of 24-h pre-incubation of F9 cells with protein solvent buffer or various concentrations of recombinant SELENOV on cell viability. (**A**) Viability cytogram of F9 cells following treatment with 100 μL/mL (equivalent to 50 μg/mL SELENOV application) or 200 μL/mL (equivalent to 100 μg/mL SELENOV application) of solvent buffer. The 10 μg/mL SELENOV group was compared to the untreated control, as the corresponding buffer volume was negligible. (**B**) Viability cytogram of F9 cells after treatment with different concentrations of recombinant SELENOV. (**C**) Effects of solvent buffer or recombinant SELENOV on cell viability and activation of cell death pathways. Data are presented as mean ± SEM. Statistical significance was determined by one-way ANOVA followed by Tukey’s post hoc test. *** *p* < 0.001 versus the Control group; ### *p* < 0.001 versus the corresponding Buffer group.

In the control group of F9 cells, 83.5% of cells remained viable, while 13.5% and 3.5% were in the early and late stages of apoptosis, respectively (Figure 3A,C). Application of 100 µL of solvent buffer did not induce cell death. However, 200 µL of solvent buffer reduced cell viability to 47%, triggering early apoptosis in 13% of cells and late apoptosis in 40%, with no detectable necrosis (Figure 3A,C). Pre-incubation of F9 cells with 10 µg/mL SELENOV significantly decreased the number of viable cells to 52%, inducing late apoptosis in 36% of cells (Figure 3B,C). Increasing SELENOV concentration to 50 µg/mL further reduced viable cells to 19%, with late apoptosis observed in 76% of cells. This dramatic effect is of particular significance as it occurred under conditions where the corresponding volume of solvent buffer (100 µL/mL) alone was non-toxic (Figure 3A,C), clearly demonstrating the potent and intrinsic cytotoxic activity of SELENOV in these cancer cells. At 100 µg/mL SELENOV, the viable cell count dropped to 5%, with late apoptosis in 66% of cells and necrosis in 17% (Figure 3B,C).

**Figure 4:**
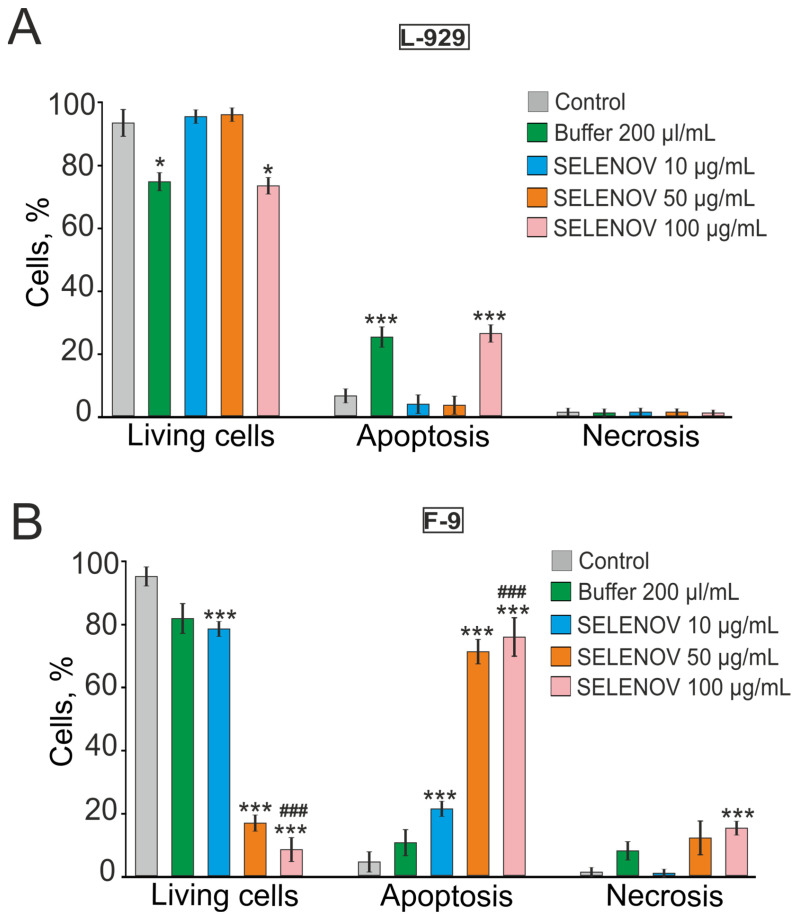
Detection of necrosis and apoptosis in L-929 (**A**) and F-9 (**B**) cells after 24-h preincubation with 200 μL/mL protein solvent buffer and various concentrations of SELENOV. Each value represents mean ± SE (*n* ≥ 3). Black asterisks indicate comparisons of experimental groups versus Control. Statistical significance was assessed using paired *t*-test; *** *p* < 0.001. Cell viability was determined using Annexin V-AF 488 Apoptosis Detection Kit (Lumiprobe). Images of stained cells are shown in Supplementary Figures S4 and S5. Data are presented as mean ± SEM. Statistical significance was determined by one-way ANOVA followed by Tukey’s post hoc test. * *p* < 0.05, *** *p* < 0.001 versus the Control group; ### *p* < 0.001 versus the corresponding Buffer group.

The method described above for detecting apoptosis in cells is routine and widely accepted; however, to more reliably identify apoptosis, we used the Apoptosis/Necrosis Detection Kit. In L-929 cells, a significant decrease in viability was observed after 24-h preincubation with 200 µL/mL of solvent buffer and SELENOV at a concentration of 100 µg/mL, due to the induction of apoptosis in 25.3% and 26.6% of cells, respectively, as detected by Annexin V-AF fluorescence (Figure 4A, Supplementary Figure S4). In F-9 cell line, a reduction in the number of viable cells occurred after preincubation with SELENOV—apoptosis was detected in 21.3% of cells at 10 µg/mL SELENOV, 71.3% at 50 µg/mL, and 76% at 100 µg/mL. Additionally, at 100 µg/mL SELENOV, necrosis was observed in 15.3% of cells, as indicated by propidium iodide fluorescence (Figure 4B, Supplementary Figure S5).

**Figure 5:**
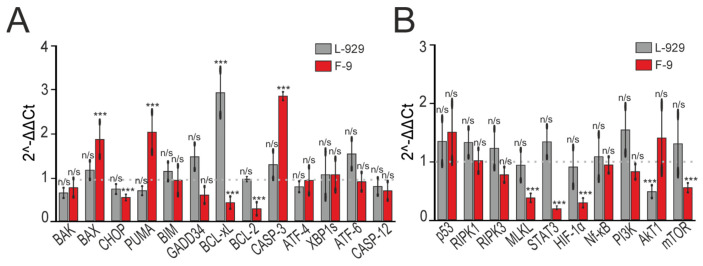
Effect of 24-h pre-incubation of L-929 and F-9 cells with 50 μg/mL SELENOV on the expression of genes encoding cell death regulatory proteins. (**A**) Changes in mRNA expression of pro- and anti-apoptotic genes following SELENOV treatment. (**B**) SELENOV-induced changes in the expression of genes involved in necroptosis, autophagy, and the STAT-3/HIF-1α signaling pathway. The dashed gray line at 1 (Control) represents the baseline expression level in cells treated for 24 h with an equivalent volume of protein solvent buffer. Statistical analysis versus Control was performed using a paired *t*-test. n/s—not significant (*p* > 0.05), *** *p* < 0.001.

Figure 5A shows changes in mRNA expression patterns of pro- and anti-apoptotic genes, along with key markers of three signaling pathways activated during endoplasmic reticulum stress. In F9 cancer cells, incubation with recombinant SELENOV was associated with significantly increased mRNA levels of several pro-apoptotic genes (BAX, PUMA, CASP-3) and decreased mRNA levels of two anti-apoptotic BCL-2 family genes (BCL-xL and BCL-2). No significant expression changes were observed for other studied genes, nor in L929 cells.

We also examined whether exogenous SELENOV affects expression of genes involved in necroptosis, autophagy, and STAT-3/HIF-1α signaling pathway. Real-time PCR results (Figure 5B) showed no statistically significant increase in mRNA levels encoding RIPK1 or RIPK3 in either cell line. However, decreased mRNA expression was observed for MLKL, STAT3, and HIF-1α in cancer cells.

### 3.3. Effects of Exogenous SELENOV on Mitochondrial Potential in Acute and Chronic Experiments

**Figure 6:**
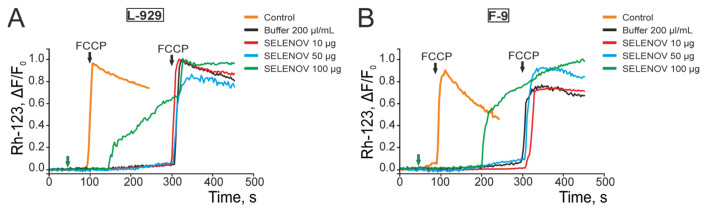
Effect of different SELENOV concentrations on the mitochondrial potential of mouse fibroblast L-929 cells (**A**) and F-9 testicular teratoma cells (**B**) in acute experiments. Control—application of 1 μM mitochondrial respiratory chain uncoupler FCCP. Buffer 200 μL/mL—application of protein solvent buffer volume equivalent to 100 μg/mL SELENOV treatment. The 10 μg/mL SELENOV group was compared to the untreated control, as the corresponding buffer volume was negligible. Experiments were performed in 6 replicates using 3 different passage cultures.

Cells were loaded with the fluorescent probe Rhodamine-123, and changes in mitochondrial membrane potential were recorded using fluorescence microscopy following treatment with various concentrations of SELENOV. Application of 200 µL/mL protein solvent buffer did not affect the mitochondrial potential of L-929 (Figure 6A) or F-9 cells (Figure 6B). In L-929 cells, treatment with 10 µg/mL or 50 µg/mL SELENOV also had no effect on mitochondrial potential, whereas 100 µg/mL SELENOV induced mitochondrial depolarization after a lag period. Subsequent application of the mitochondrial uncoupler FCCP (1 µM) caused additional depolarization (Figure 6A, green curve). In F-9 testicular teratoma cells, treatment with 10 µg/mL or 50 µg/mL SELENOV did not induce depolarization. However, 100 µg/mL SELENOV triggered strong mitochondrial depolarization, and subsequent FCCP treatment failed to induce further depolarization (Figure 6B, green curve). The inability of the protonophore FCCP to induce additional depolarization after 100 µg/mL SELENOV treatment in F-9 cells suggests profound and irreversible mitochondrial dysfunction, indicating a severe disruption of the cell’s bioenergetic capacity.

**Figure 7:**
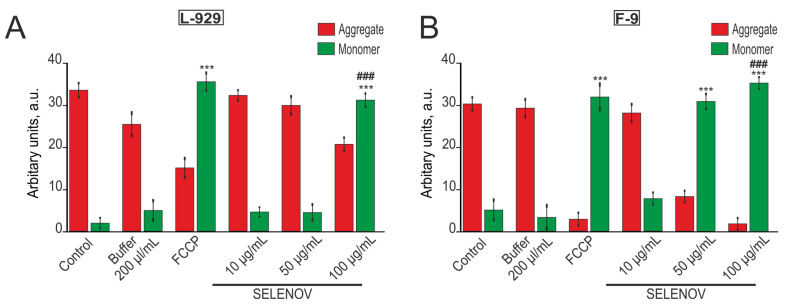
Mitochondrial membrane potential measurement by JC-1 dye in mouse fibroblast cell line L-929 (**A**) and F-9 testicular teratoma cells (**B**) after 24-h exposure to solvent buffer (200 μL/mL), respiratory chain uncoupler FCCP (1 μM), or various SELENOV concentrations. The 10 μg/mL SELENOV group was compared to the untreated control, as the corresponding buffer volume was negligible. Statistically significant differences in JC-1 monomer formation were calculated relative to the Control group. Statistical significance was determined by one-way ANOVA followed by Tukey’s post hoc test. *** *p* < 0.001 versus the Control group; ### *p* < 0.001 versus the corresponding Buffer group.

Mitochondrial depolarization was specifically detected using the cationic dye JC-1. This dye exhibits potential-dependent accumulation in mitochondria, demonstrated by a fluorescence emission shift from red to green when mitochondrial membrane potential is lost due to membrane damage. In control L-929 and F-9 cells, JC-1 remained aggregated (Figure 7, Supplementary Figure S6). Pre-incubation of both L-929 and F-9 cells with 200 µL/mL of solvent buffer for 24 h did not cause a significant increase in JC-1 monomer form. However, pre-incubation with 1 µM of the protonophore FCCP induced accumulation of JC-1 monomer form in both cell lines (Figure 7, Supplementary Figure S6). Pre-incubation of L-929 cells with 10 µg/mL or 50 µg/mL SELENOV did not result in a significant increase in JC-1 monomer form. However, addition of 100 µg/mL SELENOV to L-929 cells led to massive accumulation of JC-1 monomer form, reflecting loss of mitochondrial membrane potential and mitochondrial damage (Figure 7A, Supplementary Figure S6).

Images of stained cell cultures are shown in Supplementary Figure S6.

In F-9 cells, application of 10 µg/mL SELENOV did not cause JC-1 monomer accumulation, while treatment with 50 µg/mL or 100 µg/mL SELENOV induced similarly massive accumulation of the probe’s monomer form (Figure 7B, Supplementary Figure S6). The effect at 50 µg/mL is a robust finding, as it was triggered under conditions where the solvent control (100 µL/mL) had no effect on the mitochondrial membrane potential, unequivocally linking this dysfunction to the action of SELENOV. The disruption of mitochondrial membrane potential, including that detected by JC-1 probe, serves as a marker for early apoptosis induction, which is consistent with our above-presented data.

### 3.4. Effects of Exogenous SELENOV on ROS Production

**Figure 8:**
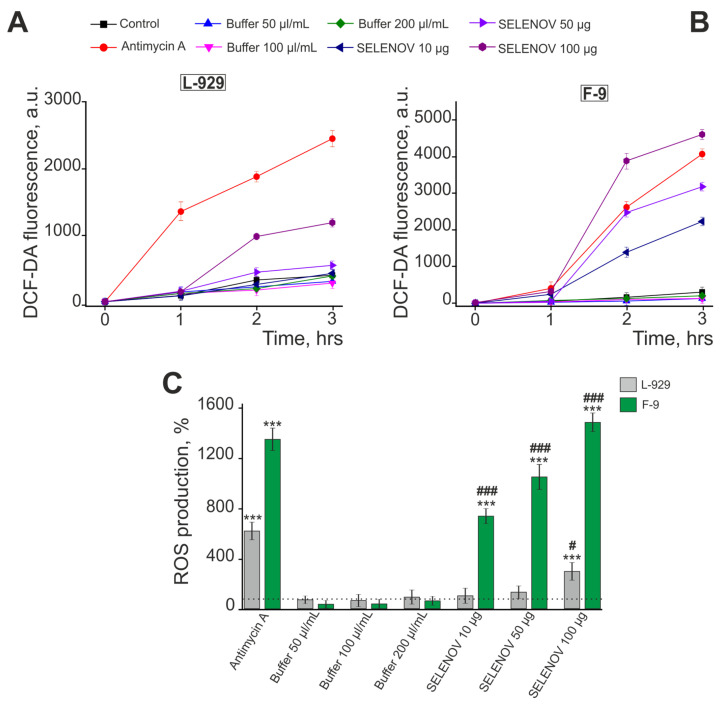
Effect of different SELENOV concentrations on ROS production in mouse fibroblast L-929 cells (**A**) and F-9 testicular teratoma cells (**B**) in acute experiments. Data are shown as mean fluorescence intensity (arbitrary units ± SEM). (**C**) ROS production in L-929 or F-9 cells expressed as percentage relative to control (dashed line) after 3-h exposure to antimycin A (20 μM), protein solvent buffer (50 μL/mL, 100 μL/mL or 200 μL/mL), or different SELENOV concentrations (10 μg/mL, 50 μg/mL, 100 μg/mL). The 10 μg/mL SELENOV group was compared to the untreated control, as the corresponding buffer volume was negligible. Statistical significance was determined by one-way ANOVA followed by Tukey’s post hoc test. *** *p* < 0.001 versus the Control group; # *p* < 0.05, ### *p* < 0.001 versus the corresponding Buffer group. The H_2_DCF-DA method measures total intracellular ROS without discriminating between specific cellular sources.

To measure ROS production, cells were cultured in 96-well plates for 24 h, loaded with the fluorescent probe H_2_DCF-DA (DCF-DA), and ROS generation was monitored using a plate reader for 3 h following treatment with various concentrations of SELENOV. In L-929 cells, only treatment with 100 μg/mL SELENOV induced increased ROS production after 2 h of exposure, while the protein solvent buffer or lower SELENOV concentrations did not affect ROS levels (Figure 8A,C).

In contrast, in F-9 cancer cells, all tested concentrations of SELENOV significantly enhanced ROS production after 2 h of treatment, whereas equivalent volumes of protein solvent buffer had no effect. The ROS overproduction induced by 50 µg/mL SELENOV is therefore uniquely attributable to the protein, as the equivalent volume of solvent buffer (100 µL/mL) did not alter ROS levels. Notably, treatment with 100 μg/mL SELENOV induced more pronounced ROS production compared to even Antimycin A (Figure 8B,C).

**Figure 9:**
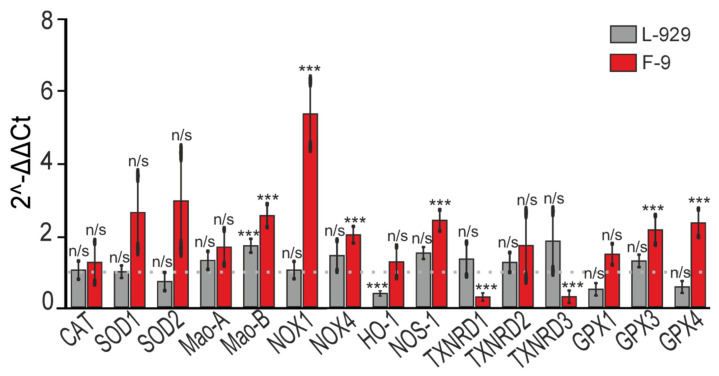
Changes in mRNA levels of genes encoding cellular redox-status proteins following 24-h pre-incubation of L-929 and F-9 cells with 50 µg/mL SELENOV. The dashed gray line at 1 (control) represents the expression level in cells treated for 24 h with an equivalent volume of protein solvent buffer. Statistical analysis of experimental groups versus Control was performed with a paired *t*-test. n/s—data not significant (*p* > 0.05), *** *p* < 0.001.

Analysis of mRNA levels of genes encoding redox-status enzymes revealed transcriptional changes in F9 cell line, SELENOV treatment was associated with significantly elevated levels of Mao-B, NOX1, and NOX4 mRNA (Figure 9). It should be noted that these data reflect changes at the transcriptional level and do not necessarily predict corresponding changes in protein activity. Furthermore, pre-incubation of these cells with SELENOV correlated with altered mRNA levels of selenium-containing thioredoxin reductases and glutathione peroxidases. The presented results demonstrate significant decrease in expression levels of TXNRD1 and TXNRD3 mRNA, as well as increase in expression levels of GPX3 and GPX4 mRNA. A trend towards increased expression is also observed for SOD1 and SOD2 mRNA. Whereas in L929 cell line, only decrease in HO-1 mRNA expression can be observed, while transcript levels of other genes did not change significantly.

### 3.5. Effects of Exogenous SELENOV on Ca^2+^ Signaling in Cells

**Figure 10:**
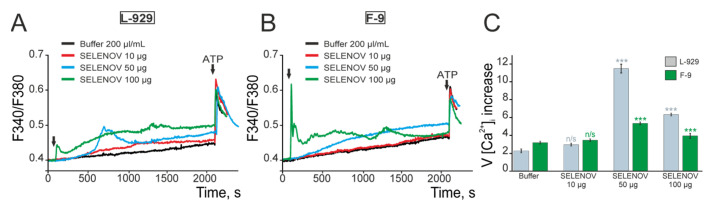
Activation of Ca2+-responses in L-929 (**A**) and F-9 cells (**B**) by different concentrations of SELENOV. Averaged Ca^2+^ signals from 50 cells are shown for each curve. (**C**)—Rate of [Ca^2+^]_i_ increase upon application of different SELENOV concentrations. The rate of [Ca^2+^]_i_ increase was measured using a linear function from the moment of SELENOV application until the end of Ca^2+^ dynamics recording. Averaged results from 3 cell cultures are presented. These results demonstrate that SELENOV can dose-dependently dysregulate calcium homeostasis. For the 50 µg/mL dose in F-9 cells, the Ca^2+^ response is a direct consequence of SELENOV application, as the solvent control at this volume (100 µL/mL) induced only a minimal increase. Buffer—application of 200 µL/mL protein solvent buffer, corresponding to the volume used for 100 µg/mL SELENOV application. Statistical significance was assessed using *t*-test. Comparison of experimental groups vs. Buffer: n/s—data not significant (*p* > 0.05), *** *p* < 0.001. Original recordings of cellular Ca^2+^ signals are presented in Supplementary Figure S7.

To identify potential effects on the Ca^2+^ signaling system in acute experiments, cell cultures were loaded with the calcium-sensitive fluorescent probe Fura-2AM. Application of the solvent buffer to L-929 cells at a volume of 200 µL/mL caused a slow increase in [Ca^2+^]_i_ over the Ca^2+^ dynamics recording period (35–38 min), as confirmed by measuring the rate of [Ca^2+^]_i_ increase using linear approximation (Figure 10). Application of 10 µg/mL SELENOV to L-929 cells did not induce a significant increase in [Ca^2+^]_i_ compared to the solvent buffer (Figure 10A,C). Application of 50 µg/mL SELENOV to L-929 cells caused a significant increase in [Ca^2+^]_i_ after a lag period, with the rate of increase being 6 times higher compared to the solvent buffer. A slight decrease in [Ca^2+^]_i_ was observed after the signal passed and a new elevated steady-state level of [Ca^2+^]_i_ was established (Figure 10A,C). Increasing the SELENOV concentration to 100 µg/mL induced a rapid rise in [Ca^2+^]_i_ in L-929 cells, establishing a new elevated steady-state [Ca^2+^]_i_ level without any downward trends (Figure 10A,C). Application of 10 µg/mL SELENOV to F-9 cells did not cause a significant increase in [Ca^2+^]_i_ compared to the solvent buffer. Application of 50 µg/mL SELENOV to F-9 cells led to a slow increase in [Ca^2+^]_i_ at a rate 2 times higher than with the solvent buffer (Figure 10B,C). Application of 100 µg/mL SELENOV to F-9 cells triggered a rapid, high-amplitude increase in [Ca^2+^]_i_, establishing a new elevated steady-state [Ca^2+^]_i_ level (Figure 10B,C).

## 4. Discussion

The obtained results demonstrate the complex effects of exogenous SELENOV on various cellular processes. The substitution of selenocysteine (Sec) with cysteine (Cys) in our recombinant SELENOV construct represents an important methodological consideration. While this substitution was necessitated by the practical challenges of heterologous selenoprotein expression in bacterial systems, which lack the specialized machinery for Sec incorporation, we acknowledge that it may alter the protein’s biochemical properties. Selenium in Sec has a lower pKa and greater nucleophilicity compared to sulfur in Cys, which can influence redox potential and catalytic efficiency [20,21]. However, several lines of evidence support the biological relevance of our findings: (1) the Cys-substituted form often serves as a functional analog for structural and mechanistic studies of selenoproteins [22,23]; (2) the conserved redox-active motif architecture is maintained; (3) our observed dose-dependent and cell-type-specific effects demonstrate that the Cys-substituted protein retains potent and specific biological activity. Future studies should aim to characterize the full-length selenoprotein form to confirm whether the effects we observed accurately reflect the native protein’s function.

Moreover, it is crucial to note that the solvent buffer used for protein delivery exhibited significant toxicity at high volumes (200 µL/mL), which complicated the interpretation of effects at the highest SELENOV dose (100 µg/mL). Therefore, the most reliable conclusions about the intrinsic bioactivity of SELENOV are drawn from the 50 µg/mL dose in F-9 cells, where the corresponding buffer volume (100 µL/mL) was non-toxic, allowing for a clear distinction between vehicle and protein effects. The observed inhibition of cell migration, particularly pronounced in testicular teratocarcinoma F-9 cells, is consistent with data on the role of selenoproteins in regulating cell motility [24]. Notably, the effect was dose-dependent and more prominent in tumor cells compared to normal L-929 fibroblasts, which aligns with findings by Chen et al. [9]. This may suggest selective action of the protein on transformed cells, representing particular interest for potential antitumor applications.

The cytotoxic effects of SELENOV were primarily mediated through apoptosis induction, with substantial differences in sensitivity between cell lines. In F-9 cells, apoptosis was triggered at relatively low protein concentrations, whereas L-929 cells required higher doses for comparable effects. The observed changes in mRNA levels of apoptosis-regulating genes are consistent with the observed apoptotic phenotype and with known mechanisms of selenoprotein action, potentially involving modulation of the mitochondrial cell death pathway [25]. Importantly, the robust induction of late apoptosis in 76% of F-9 cells by 50 µg/mL SELENOV occurred under conditions where the solvent control was non-toxic, providing unequivocal evidence of the protein’s direct pro-apoptotic action.

An important aspect of SELENOV’s action is its effect on mitochondrial function. The observed depolarization of the mitochondrial membrane, particularly pronounced in tumor cells [26], correlates with apoptosis activation and may be considered a key element in the mechanism of the protein’s cytotoxic action [27]. Interestingly, in F-9 cells, the mitochondrial potential did not recover after exposure to high SELENOV concentrations, indicating profound disturbances in mitochondrial function, which is a primary prerequisite for an energetic crisis in the cell. A key question arising from our study is the primary site of exogenous SELENOV action—whether it initiates signaling at the cell surface or exerts its effects intracellularly. While our experimental design was focused on characterizing the phenotypic consequences of SELENOV treatment rather than tracing its cellular localization, the nature of the observed effects provides strong clues. The rapid induction of sustained intracellular Ca^2+^ elevation and ROS production could be consistent with initial signaling events at the plasma membrane, potentially through an unidentified receptor. However, the profound execution of intrinsic apoptosis, characterized by mitochondrial depolarization and the transcriptional modulation of core BCL-2 family genes (BAX, PUMA, BCL-2), strongly suggests that the functional consequences of SELENOV are mediated intracellularly. We hypothesize that the protein is likely internalized, possibly via receptor-mediated endocytosis, to directly or indirectly engage mitochondrial pro-apoptotic machinery. Elucidating the precise subcellular localization of internalized SELENOV and identifying its direct binding partners represent crucial objectives for future research.

Concurrently with mitochondrial changes, we observed increased production of reactive oxygen species (ROS) upon exposure to exogenous SELENOV, especially prominent in tumor cells. This effect was accompanied by altered expression of genes encoding antioxidant defense enzymes and oxidative stress generators. It is well established that changes in ROS metabolism and redox signaling are frequently observed in cancer cells and may play important roles in tumor development and drug resistance [28,29]. Since many cancer cells inherently maintain higher ROS levels compared to normal cells, they exist under oxidative stress and are more sensitive to additional ROS stress. Our work demonstrates that adding exogenous SELENOV to culture medium increases total intracellular ROS levels. Concurrently, we observed transcriptional upregulation of Mao-B, NOX1, and NOX4 mRNA. While these genes are implicated in ROS metabolism, our data do not establish a causal relationship between their transcriptional changes and the observed ROS increase. Future studies measuring protein expression and enzymatic activity are needed to confirm their functional role in this context.

Available data suggest that two monoamine oxidases may have opposing roles in cancer progression modulation. In prostate cancer, Mao-A plays an oncogenic role by promoting tumor development and metastasis [30], while Mao-B activity decreases in prostate cancer tissues, correlating with favorable clinical outcomes. In vitro studies have shown that elevated Mao-B expression suppresses cancer cell proliferation and motility [31]. The SELENOV-induced increase in Mao-B mRNA represents a correlative observation, and its potential functional contribution to the anticancer effects remains to be tested in future loss-of-function experiments.

It is known that imbalance in NADPH oxidase (Nox) enzyme activity may potentially contribute to cancer progression, as these enzymes have been repeatedly shown to be essential for survival and growth of human cancer cells [32,33,34,35]. This makes it difficult to explain the observed increase in their mRNA expression alongside activation of apoptotic death in F-9 cancer cells. However, it should be noted that in acute experiments, exogenous SELENOV caused substantial ROS increase in F-9 cancer cells, suggesting the resulting ROS imbalance may cause uncontrolled expression of pro-oxidant enzyme genes.

In this study, we also examined changes in mRNA expression of three selenium-containing thioredoxin reductases and four glutathione peroxidases, with particular focus on TXNRD3 and GPX4, as these two enzymes—along with SELENOV—are specifically localized in testes [36]. Our results demonstrate that exogenous SELENOV downregulated TXNRD1 and TXNRD3 mRNA expression, which may represent an important anticancer effect since both enzymes are frequent pharmacological targets in various cancer therapies [37,38,39]. The upregulation of GPX3 mRNA following SELENOV treatment may also be associated with suppression of F9 cancer cell proliferation. GPX3 has been identified as a tumor suppressor in multiple cancers, where epigenetic regulation of its mRNA expression through hypermethylation of CpG islands in the promoter region specifically reduces transcriptional efficiency in many mammalian cancers [40,41]. Conversely, GPX4 is known to act as an oncogene that inhibits ferroptosis in cancer cells and reduces their drug resistance to established anticancer agents [42,43,44,45]. We also observed SELENOV-induced changes in the mRNA levels of glutathione peroxidases GPX3 and GPX4. GPX4 is a key regulator of ferroptosis, and its modulation can influence cell fate. However, the transcriptional changes reported here are preliminary and do not allow us to conclude whether ferroptosis is activated or suppressed in our model. The role of these selenoproteins, along with the potential contribution of ferroptosis to SELENOV’s cytotoxic effect, remains an open question that requires direct investigation of protein levels, lipid peroxidation, and other functional markers in future studies.

Analysis of mRNA levels in F9 cells revealed that SELENOV treatment was accompanied by upregulation of several pro-apoptotic genes. These findings are supported by viability assays (Figure 3 and Figure 4B) clearly showing high percentages of cancer cells undergoing apoptosis after treatment with 50 μg/mL SELENOV. However, we observed no statistically significant changes in expression levels of key markers from the three UPR (unfolded protein response) pathways activated during ER stress, suggesting this process likely does not contribute to the apoptotic death of these cancer cells. It is noteworthy that other selenium-containing compounds, such as methylseleninic acid, can induce apoptosis specifically through ER stress activation [46], highlighting the diversity of molecular mechanisms among selenium-based agents. While this correlation is consistent with the activation of the intrinsic apoptotic pathway, further mechanistic studies are needed to establish a direct causal relationship between these specific transcriptional changes and cell death execution.

Some results were obtained for key regulators of necroptosis—RIPK1/RIPK3/MLKL. The absence of increased mRNA expression of necroptosis markers should certainly be viewed positively, as this process is frequently implicated in cancer progression [47]. In this work, we also monitored changes in mRNA expression patterns of signal transducer and activator of transcription-3 (STAT3) and hypoxia-inducible factor 1α (HIF-1α). STAT3 is known to inhibit degradation and enhance synthesis of HIF-1α. These proteins cooperate to activate HIF-1α target genes and promote HIF1-dependent tumorigenesis under hypoxic conditions [48,49]. The observed significant suppression of STAT3 and HIF-1α mRNA levels in F9 cancer cells (absent in fibroblasts) correlates with the anti-cancer effects of exogenous SELENOV and may represent an important aspect of its therapeutic potential. These findings align with the concept of dual roles for selenoproteins, which can function as either antioxidants or pro-oxidants depending on concentration [27,50].

The observed alterations in calcium signaling induced by SELENOV are particularly interesting given calcium’s crucial role in apoptosis regulation and cellular homeostasis. The more pronounced response of tumor cells to the protein may reflect distinctive features of calcium metabolism in transformed cells [10,51] and warrants further investigation.

Comparative analysis of SELENOV effects in normal and tumor cells revealed an important feature—selective action on transformed cells. This selectivity may be associated with fundamental differences in redox homeostasis, energy metabolism and apoptosis regulation between normal and tumor cells [52,53]. The obtained results open prospects for further study of SELENOV as a potential antitumor agent, but require additional research to clarify its molecular mechanisms of action and evaluate possible therapeutic potential. A key methodological limitation of the present study is the toxicity of the protein solvent buffer at high volumes (200 µL/mL), which confounds the effects of the highest SELENOV dose (100 µg/mL). While we have controlled for this by using buffer-only groups and have based our core conclusions on doses where the vehicle was non-toxic (e.g., 50 µg/mL in F-9 cells), this issue underscores the importance of developing alternative delivery formulations for future in vivo studies.

Limitations and Future Directions. This study has several limitations that should be acknowledged. First, the mechanistic interpretations are primarily based on transcriptional data (mRNA levels). While the observed changes in gene expression—including genes in the BCL-2 family, NOX enzymes, and selenoproteins such as GPX4—are statistically significant and consistent with functional outcomes like apoptosis and increased ROS, they remain correlative. The magnitude of these changes was relatively modest (2–3 fold), and they do not constitute proof of direct mechanistic involvement or establish causality. The absence of protein-level validation, for example, via Western blotting or activity assays, significantly limits mechanistic interpretation. This gap is partly attributable to the current lack of commercially available, well-validated antibodies against SELENOV. Therefore, future work should incorporate proteomic approaches, custom antibody development, and direct measurement of pathway activities—such as lipid peroxidation for assessing ferroptosis—to verify the protein-level consequences of SELENOV treatment and confirm the proposed mechanisms.

Second, the experimental design does not establish causality between specific transcriptional changes and cell death. Future studies employing genetic tools (e.g., siRNA or CRISPR knockdown/knockout) and biochemical assays will be essential to definitively elucidate the molecular mechanisms underlying SELENOV’s selective pro-apoptotic action and to test the functional roles of pathways discussed here, such as those involving STAT3/HIF-1α or Mao-B.

Third, a notable methodological constraint is the toxicity of the protein solvent buffer at high volumes (200 µL/mL), which complicates the interpretation of the highest SELENOV dose (100 µg/mL). Although controlled for via buffer-only groups, this issue highlights the need to develop alternative, non-toxic delivery formulations for future in vivo studies.

Finally, the potential translation of SELENOV as a therapeutic agent faces broader challenges inherent to protein-based therapies. Key obstacles include low stability and short half-life of proteins in biological environments due to rapid proteolytic degradation, often requiring frequent administration or advanced delivery systems (e.g., polymer nanocapsules, liposomes) to maintain effective concentrations [54,55]. Another challenge is immunogenicity, where recombinant proteins may induce humoral immune responses and neutralizing antibodies, potentially reducing efficacy and causing adverse reactions [56]. This risk is particularly relevant for SELENOV, as even minor differences between the recombinant and endogenous protein could enhance immunogenicity. It is also important to consider limited bioavailability and poor tissue penetration across biological barriers such as the blood–brain barrier and dense tumor stroma, which can restrict delivery to target sites [57,58]. However, studies of other selenium-containing agents, such as selenium nanoparticles, demonstrate promising strategies to overcome these limitations and confirm the therapeutic potential of modulating selenium-dependent pathways in oncology [59]. Furthermore, there is potential on-target toxicity in normal cells, as suggested by our observation that high SELENOV concentrations also increase ROS and cause mitochondrial depolarization in L-929 fibroblasts, underscoring the need for careful therapeutic dose selection [60].

Thus, while our in vitro findings highlight the promising selective cytotoxicity of SELENOV, translating this protein into clinical practice will require addressing these fundamental and technological challenges through further interdisciplinary research.

## 5. Conclusions

Exogenous SELENOV exhibits a potent pro-apoptotic function, as decisively demonstrated by its action at 50 µg/mL in F-9 testicular teratoma cells, where its effects are clearly distinguishable from a non-toxic vehicle control. Its effects are mediated through mitochondrial dysfunction (evidenced by membrane depolarization), overall increase in cellular ROS levels, and Ca^2+^ dysregulation. These findings underscore SELENOV’s potential as a modulator of cellular stress responses and its context-dependent cytotoxicity, warranting further exploration for therapeutic applications in oncology.

## Figures and Tables

**Table 1:** Sequences of oligonucleotides (mouse) used in the real-time PCR reaction.

Gene Names	Forward Primer 5′->3′ Revers Primer 5′->3′
*GAPDH*	CGACTTCAACAGCAACTCCC GTGTCCTTGCTGGGGTGGG
*RIPK1*	AAGGAGCCCTATGAGAATGTC ACATCCTCTTCTACATATTCTTC
*RIPK3*	TTCGATGGCCCAACCTCCC TGCCCGAAGGTGCCAAGCC
*MLKL*	CAAAGAGCACTAAAGCAGAGAG GGCAATCCTGACCCACTGG
*Nf-κB*	AAGTGCAAAGGAAACGCCAGAA ACTACCGAACATGCCTCCACCA
*GADD34*	GAGTCCCATGAAGAGATTGTAC ACCAGCCCAGCAGCACTTAG
*p53*	CTTCAGATCCGTGGGCGTG CAAGAAGTGGAGAATGTCAGTC
*STAT3*	CCCCGTACCTGAAGACCAAG ATGGGGTTCGGCTGCTTAGG
*Bcl-2*	CTACGAGTGGGATGCTGGAGATG TCAGGCTGGAAGGAGAAGATGC
*Bax*	TAAAGTGCCCGAGCTGATCAGAAC CTTCCCAGCCACCCTGGTCTT
*Bim*	GATCCTCCCTGCTGTCTCG AGGCGGACAATGTAACGTAAC
*Bak*	CAGATGGATCGCACAGAGAG GCGTCTTTGCCCTGGGGAG
*Chop*	CAGCTGGGAGCTGGAAGCCTG GACCACTCTGTTTCCGTTTCC
*Puma*	TCTCGGGGGCTCTGTGGC AGCACCTGGAGTCGCCCG
*Casp-3*	CTCTTCATCATTCAGGCCTGC GACCCGTCCTTTGAATTTCTC
*HIF-1α*	GGCGACTGTGCACCTACTATG TGATCCAAAGCTCTGAGTAATTC
*BCL-xL*	TGGCCACAGCAGCAGTTTG TCTCCGGTACCGCAGTTCAA
*ATF-4*	TCGGGTTTGGGGGCTGAAG AAACAGAGCATCGAAGTCAAAC
*ATF-6*	AGGAGGGGAGATACGTTTTAC CGAGGAGCTTTTGATGTGGAG
*Casp-12*	GGCTCTATCTTCATCACACAAC CTGGGCTGCTTGTGGCTTC
*XBP1s*	AGTCCGCAGCACAGCAGGT AGAGAAAGGGAGGCTGGTAAG
*mTOR*	AGATAAGCTCACTGGTCGGG CTGTCTGTCCATGGTTTTAGT
*AKT1*	ATGTGTATGAGAAGAAGCTGAG GCGGGGCTTCTGGACTCGG
*PI3K*	TGGCTGGGGAATGAAAATACC AGGGAGCTGTACAGGTTGTAG
*TXNRD2*	ACTATGGCTGGGAGGTGGC CCTTTGACTTGTGTGGGGTAC
*TXNRD3*	CTCTTTAGAAAAGTGTGATTATATT GCCCACATTTCATTGCAGCTG
*GPX1*	GGGGAGCCTGTGAGCCTGG GGACGTACTTGAGGGAATTC
*GPX3*	GAAAGGAGATGTGAACGGGG GTGGGGGCATCAGTTACTTC
*GPX4*	GATGAAAGTCCAGCCCAAGG GAAGGCTCCAGGGGTCACAG
*Nos-1*	GCTGCAGGTGTTCGATGCCC CCAAGGTAGAGCCATCTGGCTGCTT
*HO-1*	AGGGTGACAGAAGAGGCTAAG AATTCCCACTGCCACTGTTGC
*Cat*	GCTGACACAGTTCGTGACCCTCG ACAGGCAAGTTTTTGATGCCCTGGT
*Mao-A*	GGCGGCATCTCAGGATTGGCT TATGCCAAGGGGTTCCACACAGGT
*Mao-B*	GCCTCAGTGTGGTGGTTCTGGAAG CACTGGGAATCTCTTGGCCCATCTCATC
*SOD1*	TCGAGCAGAAGGCAAGCGGTG CGGCCAATGATGGAATGCTCTCCTGAG
*SOD2*	CTCCCGGCACAAGCACAGC TCCTTTGGGTTCTCCACCACCCTT
*NOX1*	CCATTTGACAATGGGAAACTGGCTGGTT ACCAGCTTATGGAAGGTGAGGTTGTGAT
*TXNRD1*	CAACAAATGTTATGCAAAAATAATC ACACTGGGGCTTAACCTCAG
*NOX4*	GTCCTGGAGGAGCTGGCTGG CTTGTTCTCCTGCTAGGGACCTTCTGTG

## Data Availability

The original contributions presented in this study are included in the article/Supplementary Materials. Further inquiries can be directed to the corresponding author.

## Supplementary Materials

The following supporting information can be downloaded at https://www.mdpi.com/article/10.3390/biom15121733/s1, Figure S1: Expression and purification of recombinant mSELENOV (Sec → Cys). Figure S2: Images from scratch-wound healing assays performed on F-9 cells at 0, 24, and 48 h post-treatment. Figure S3: Images from scratch-wound healing assays performed on F-9 cells at 0, 24, and 48 hours post-treatment.Figure S4: Effect of a 24-hour incubation of L-929 cells with the protein solvent buffer (200 µL/mL) and different concentrations of SELENOV on the induction of apoptosis and necrosis. Figure S5: Effect of a 24-hour incubation of F-9 cells with the protein solvent buffer (200 µL/mL) and different concentrations of SELENOV on the induction of apoptosis and necrosis.Figure S6: Measurement of mitochondrial membrane potential in L-929 and F-9 cells using JC-1 fluorescent probe staining. Figure S7: Ca^2+^ signals in L-929 and F-9 cell lines following application of 200 μL/mL protein solvent buffer and various concentrations of SELENOV.
