# Supplementary material for: Exogenous Selenoprotein V Induces Apoptosis in Murine Testicular Teratoma Cells via Mitochondrial Dysfunction and ROS Overproduction
Source: Biomolecules. 2025 Dec 12;15(12):1733. doi: 10.3390/biom15121733 (PMC12730894; doi:10.3390/biom15121733)
Supplement: Supplementary file 1 [file biomolecules-15-01733-s001.zip › biomolecules-4008672-supplementary.pdf]

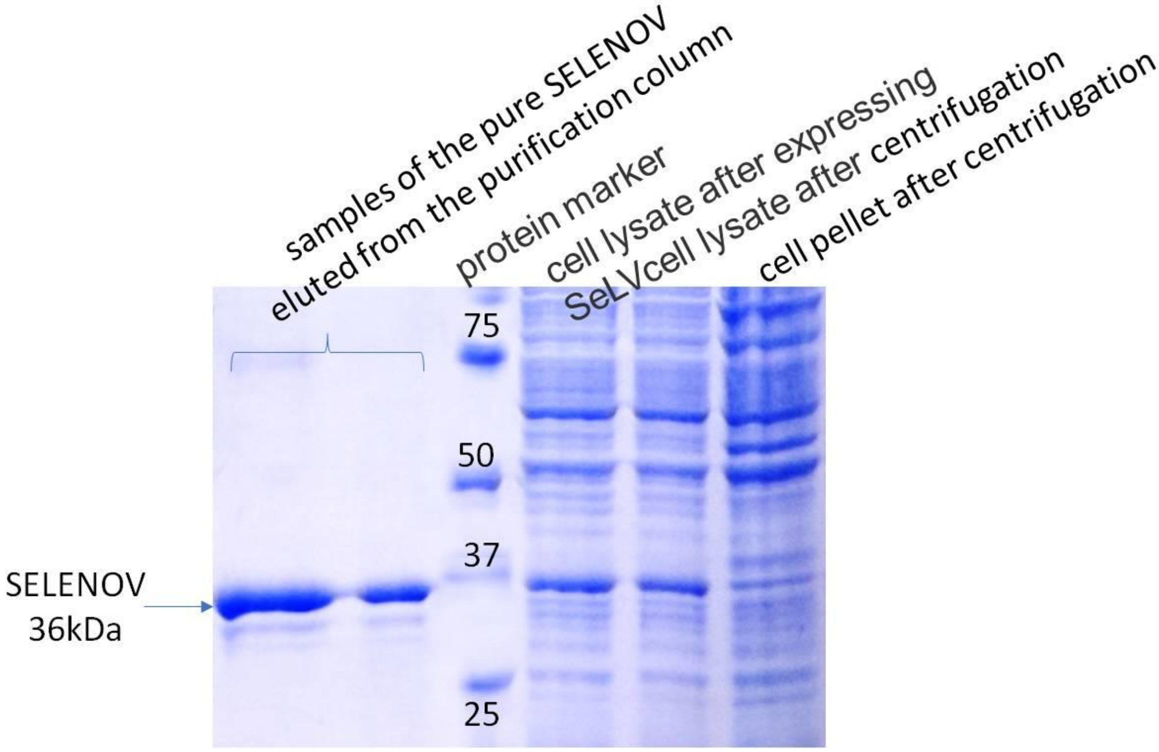

Supplementary, Figure S1. Expression and purification of recombinant mSELENOV (Sec→Cys).

Top: Schematic representation of the SDS-PAGE analysis. Bottom: Coomassie-stained 12.5% polyacrylamide gel. Lanes: M – protein molecular weight marker (sizes indicated in kDa); 1 – total cell lysate after induction of SELENOV expression with IPTG; 2 – insoluble fraction (pellet) after centrifugation of the lysate; 3 – soluble fraction (supernatant) after centrifugation; 4 – purified recombinant mSELENOV (Sec→Cys) eluted from the nickel-agarose affinity column. The dominant band at approximately 36 kDa (indicated by the arrow) corresponds to the His-tagged recombinant protein. The presence of SELENOV primarily in the soluble fraction (lane 3) and its high purity after affinity chromatography (lane 4) are demonstrated.

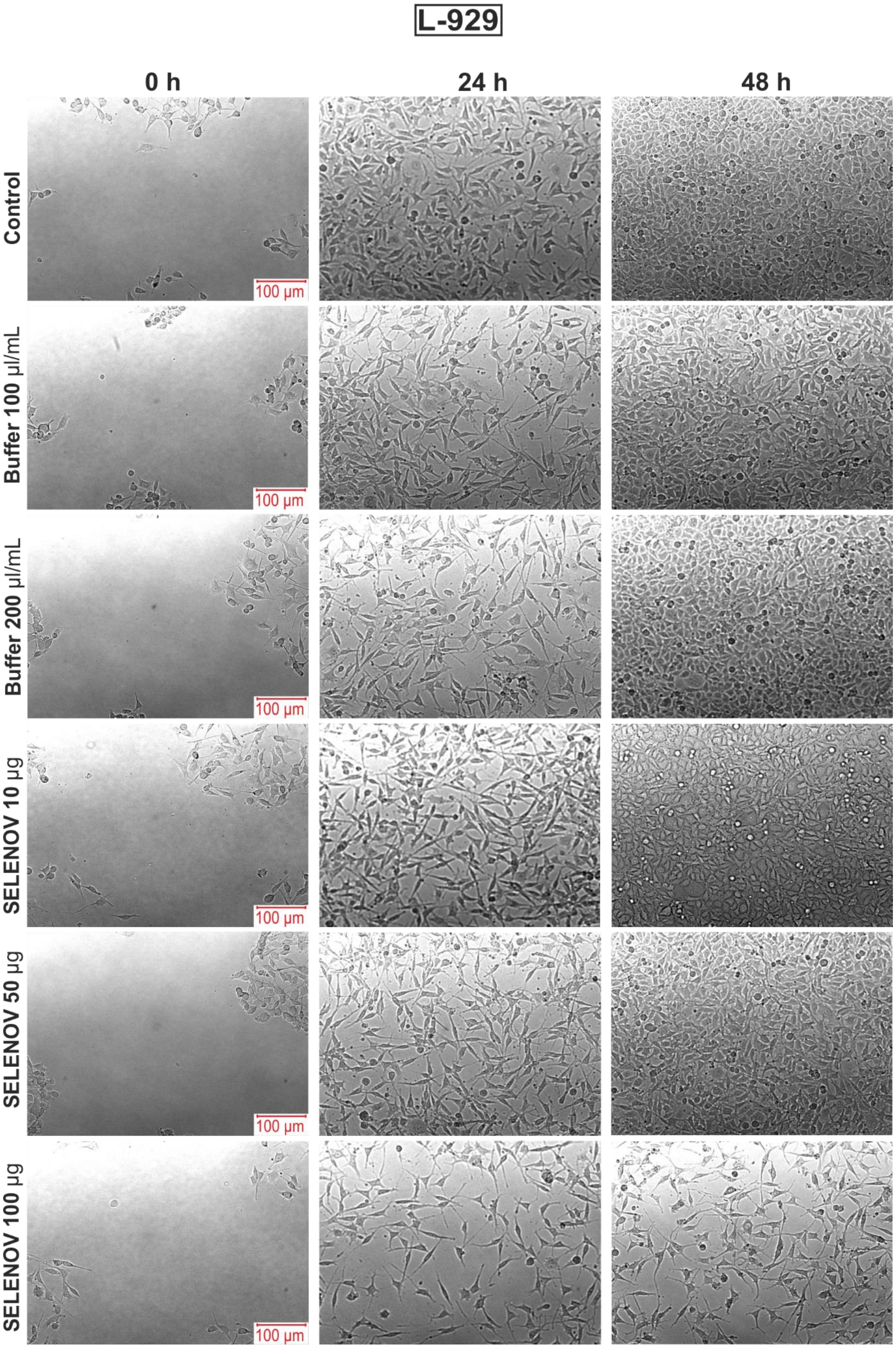

Supplementary, Figure S2. Images from scratch-wound healing assays performed on L-929 cells at 0, 24, and 48 hours post-treatment. Cells were treated with the protein solvent buffer at a volume of 200 µL/mL, corresponding to the addition of 100 µg

SELENOV and various concentrations of SELENOV. The experiment was repeated three times, and representative images are shown. Images were captured using an inverted Carl Zeiss microscope at 10× magnification.

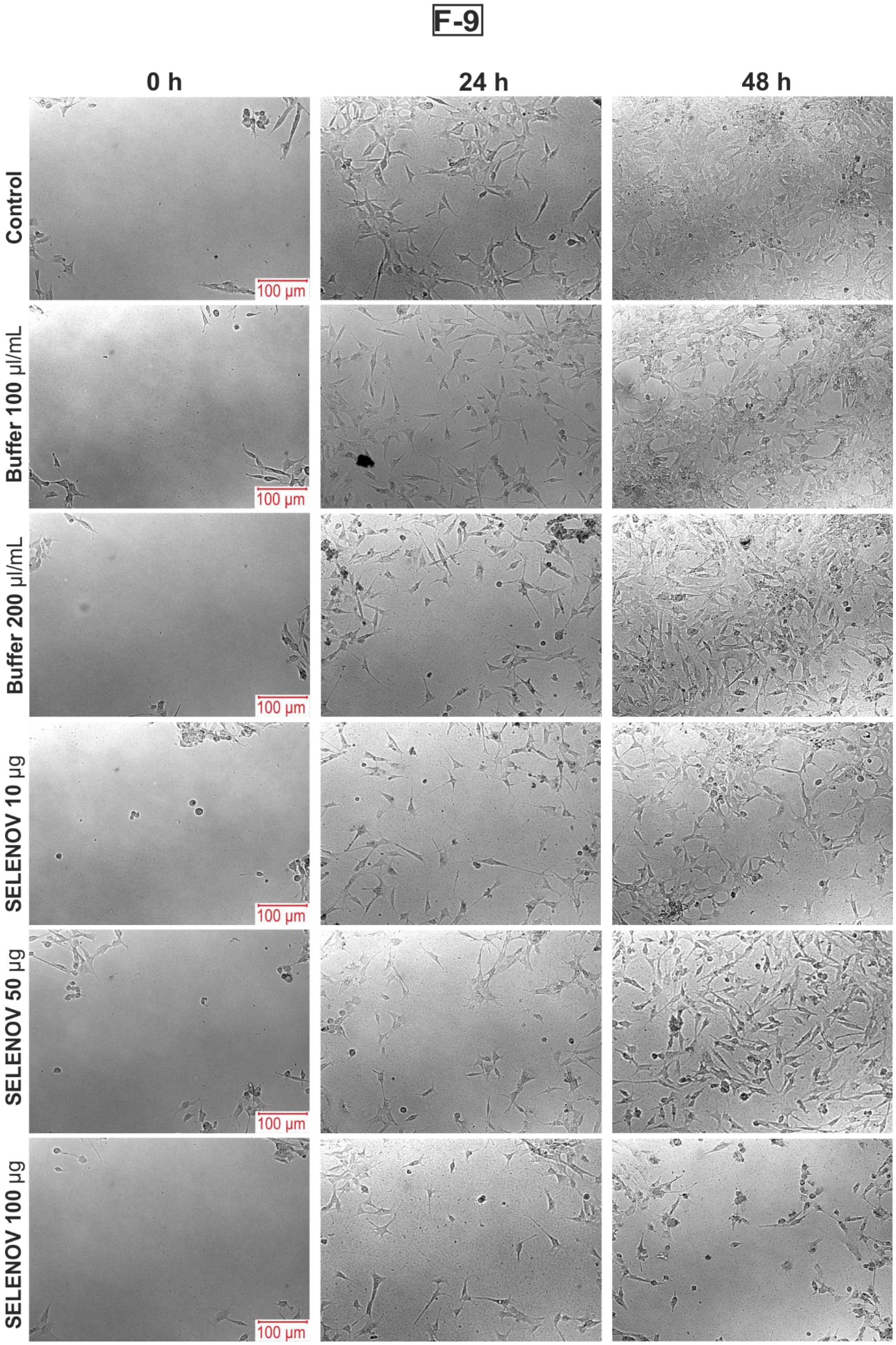

Supplementary, Figure S3. Images from scratch-wound healing assays performed on F-9 cells at 0, 24, and 48 hours post-treatment. Cells were treated with the protein solvent buffer at a volume of 200 µL/mL, corresponding to the addition of 100 µg SELENOV

and various concentrations of SELENOV. The experiment was repeated three times, and representative images are shown. Images were captured using an inverted Carl Zeiss microscope at 10× magnification.

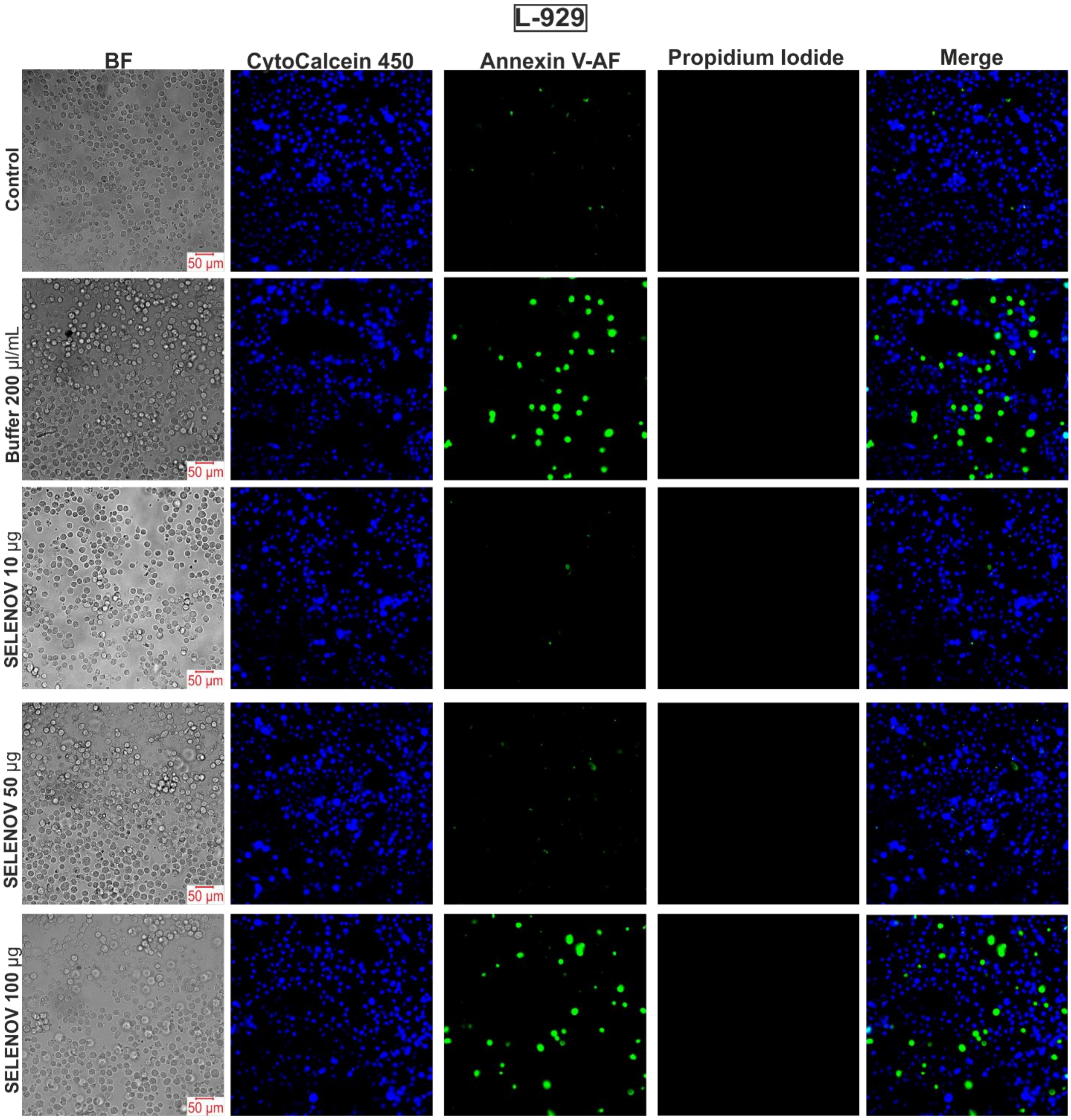

Supplementary, Figure S4. Effect of a 24-hour incubation of L-929 cells with the protein solvent buffer (200 µL/mL) and different concentrations of SELENOV on the induction of apoptosis and necrosis. Cell staining was performed using an Apoptosis/Necrosis Detection Kit. Abbreviations: BF – bright-field microscopy; CytoCalcein – marker for viable cells; Annexin V-AF – apoptotic cells marker; Propidium iodide – necrotic cells marker.

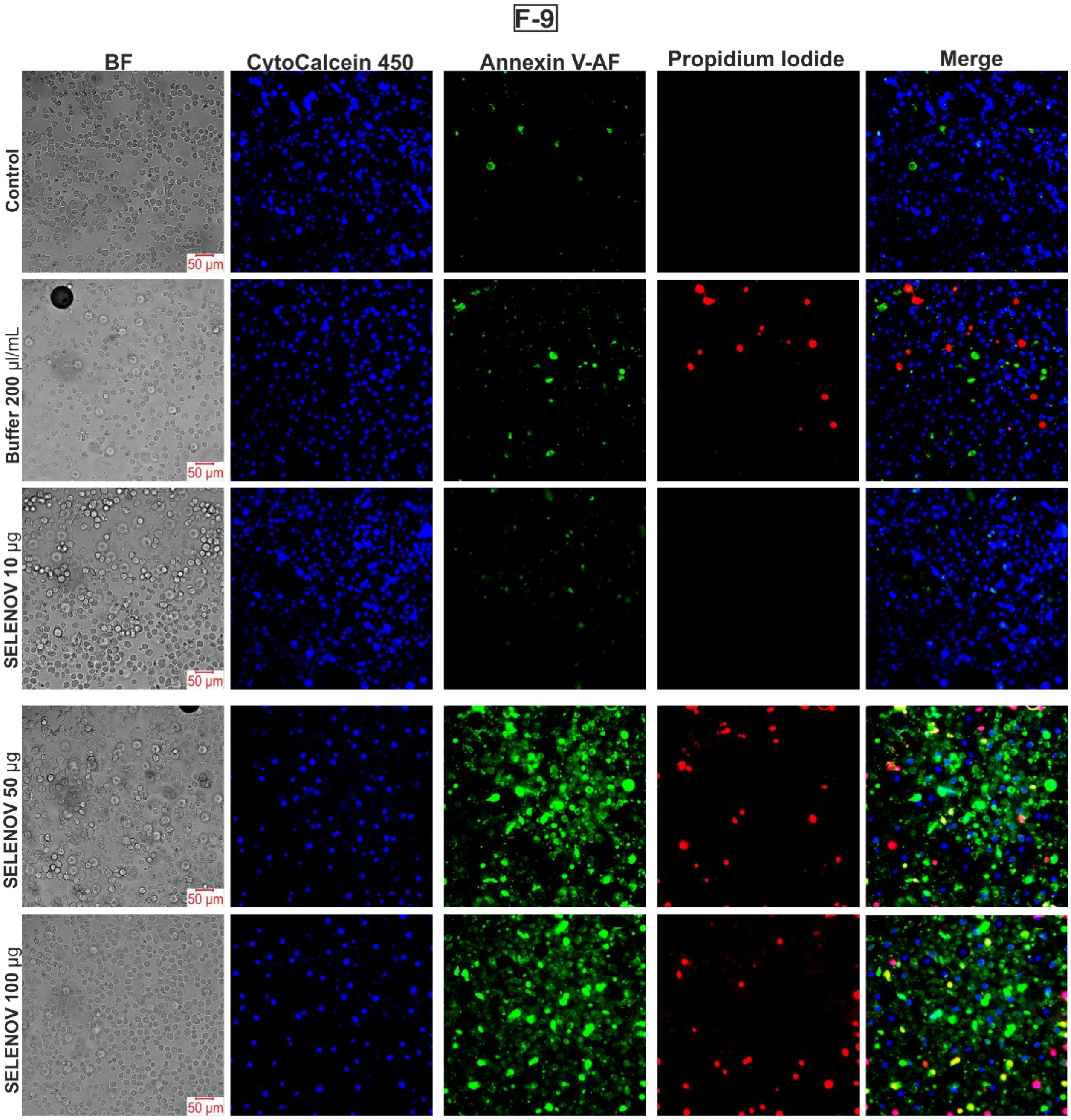

Supplementary, Figure S5. Effect of a 24-hour incubation of F-9 cells with the protein solvent buffer (200 µL/mL) and different concentrations of SELENOV on the induction of apoptosis and necrosis. Cell staining was performed using an Apoptosis/Necrosis Detection Kit. Abbreviations: BF – bright-field microscopy; CytoCalcein – marker for viable cells; Annexin V-AF – apoptotic cells marker; Propidium iodide – necrotic cells marker.

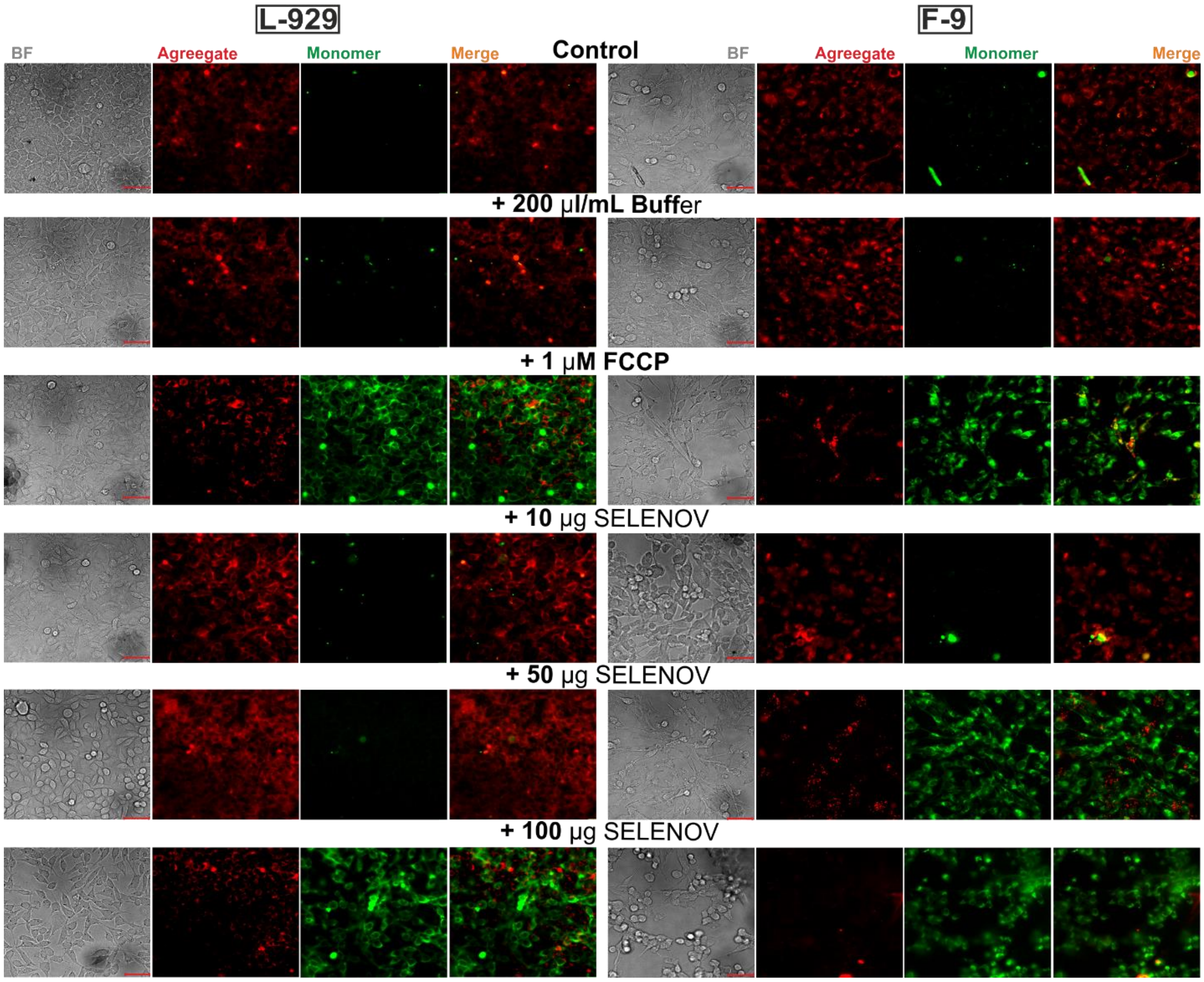

Supplementary, Figure S6. Measurement of mitochondrial membrane potential in L-929 and F-9 cells using JC-1 fluorescent probe staining. Cells were treated with protein solvent buffer (200 µL/mL), 1 µM FCCP, and various concentrations of SELENOV for 24 hours before fluorescence detection. Scale bar: 50 µm.

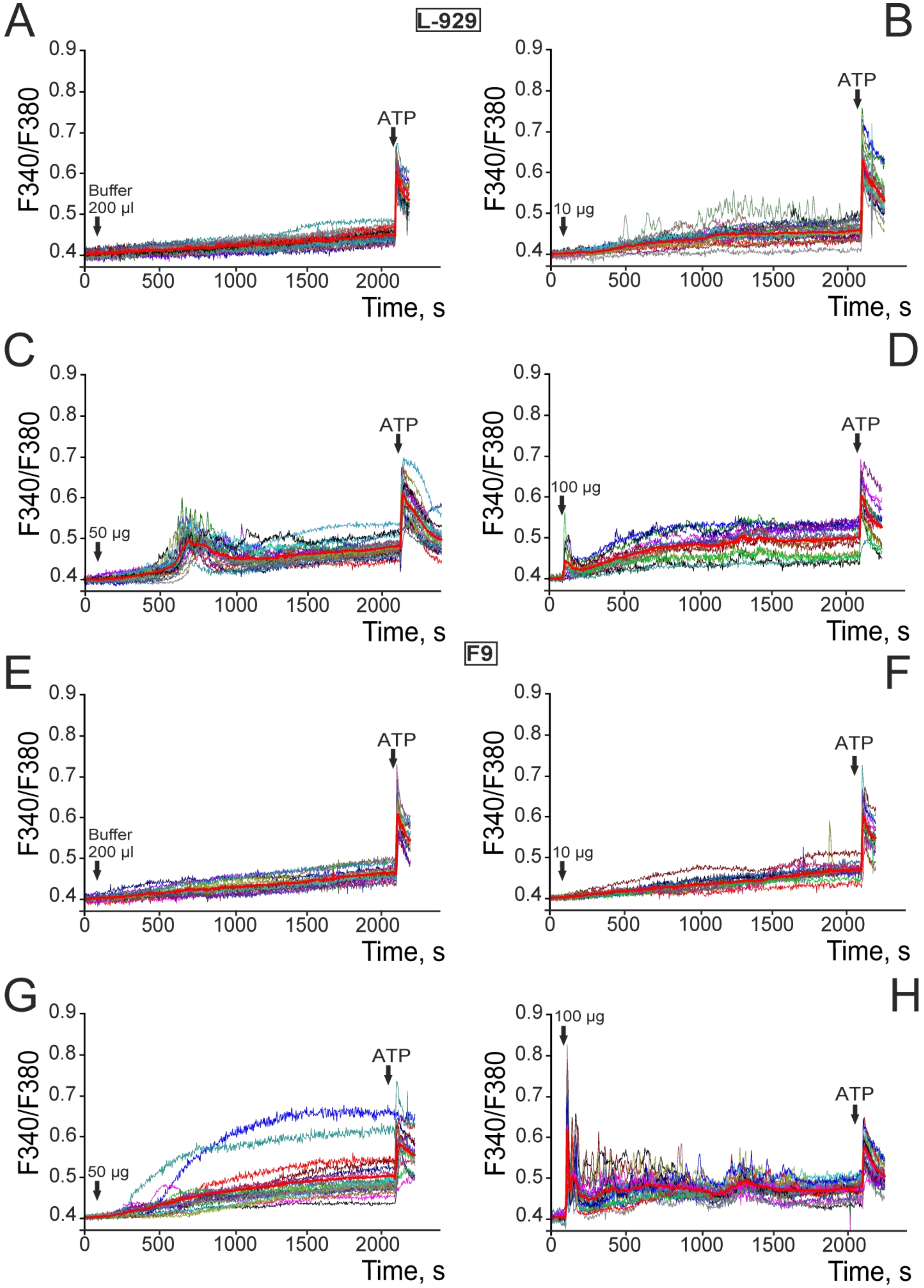

Supplementary, Figure S7. Ca^{2+} signals in L-929 and F-9 cell lines following application of 200 μL/mL protein solvent buffer and various concentrations of SELENOV. The

figure shows Ca^{2+} responses of cells in a single experiment and their average value (thick red curve).
